# Immunomodulatory role of metalloproteinase ADAM17 in tumor development

**DOI:** 10.3389/fimmu.2022.1059376

**Published:** 2022-11-17

**Authors:** Kai Wang, Zixue Xuan, Xiaoyan Liu, Meiling Zheng, Chao Yang, Haiyong Wang

**Affiliations:** ^1^ Key Laboratory of Epigenetics and Oncology, Research Center for Preclinical Medicine, Southwest Medical University, Luzhou, China; ^2^ Clinical Pharmacy Center, Department of Pharmacy, Zhejiang Provincial People’s Hospital, Affiliated People’s Hospital, Hangzhou Medical College, Hangzhou, China; ^3^ National Engineering Research Center for Marine Aquaculture, Institute of Innovation & Application, Zhejiang Ocean University, Zhoushan, China; ^4^ Department of Internal Medicine Oncology, Shandong Cancer Hospital and Institute, Shandong First Medical University and Shandong Academy of Medical Sciences, Jinan, China

**Keywords:** ADAM17, tumor microenvironment, shedding activity, immune response, inflammation

## Abstract

ADAM17 is a member of the a disintegrin and metalloproteinase (ADAM) family of transmembrane proteases involved in the shedding of some cell membrane proteins and regulating various signaling pathways. More than 90 substrates are regulated by ADAM17, some of which are closely relevant to tumor formation and development. Besides, ADAM17 is also responsible for immune regulation and its substrate-mediated signal transduction. Recently, ADAM17 has been considered as a major target for the treatment of tumors and yet its immunomodulatory roles and mechanisms remain unclear. In this paper, we summarized the recent understanding of structure and several regulatory roles of ADAM17. Importantly, we highlighted the immunomodulatory roles of ADAM17 in tumor development, as well as small molecule inhibitors and monoclonal antibodies targeting ADAM17.

## Introduction

Transmembrane proteolysis is a post-translational modification that plays an important role in cellular biological processes, such as signal transduction and immune responses (1–3). Many transmembrane proteins need to be cleaved from the cell surface and released in a soluble form to initiate cellular or intercellular signal transduction (4–6). ADAM17, also known as tumor necrosis factor (TNF)-α converting enzyme (TACE), CD156B, NISBD1, and snake venom-like protease (cSVP), is a member of the disintegrin and metalloprotease family. ADAM17 exists in two forms: precursor and activated ADAM17. Activation of ADAM17 is required for the cleavage of its prodomain and exposure of the active site. In response to the inflammatory stimuli, activated ADAM17 prompts multiple receptor-mediated signal transduction by cleaving ectodomains of membrane proteins, including inflammatory cytokines, growth factors, receptors, and adhesion factors (7). The expression of ADAM17 in mouse articular cartilage is positively correlated with the development of arthritis, and its deletion attenuates articular cartilage degeneration (8). Moreover, ADAM17 is associated with glomerular inflammation and fibrosis (9). In diabetic mice, ADAM17 deletion in the proximal tubules improves glucose tolerance, prevents podocyte loss, and inhibits the accumulation of glomerular macrophages and collagen (9). More importantly, ADAM17 is contributory to the occurrence and development of cancers, including lung carcinoma (10), ovarian carcinoma (11), breast carcinoma (12–14), gastric carcinoma (15), and cervical carcinoma (16). Interestingly, it regulates some immune signaling pathways through the shedding activity, which may facilitate the inflammatory response in tumor development (17–19). However, the study of the relationship between the abnormal expression of this metalloproteinase in tumors and its immune regulation is still not well studied. Herein, we summarized and updated multiple regulatory roles of ADAM17 as well as the development of ADAM17 inhibitors with a focus on the immunomodulatory role of ADAM17 in tumor development, which may provide reasonable insights for the prevention and treatment of cancer diseases.

## Characterization of ADAM17

ADAM17 is a widely distributed transmembrane protein that is involved in different physiological processes such as inflammation, cell proliferation and apoptosis by its hydrolysis of various precursor membrane proteins, such as TNF-α, TNFRII, HB-EGF, IL-1R1, etc. It is localized in the membranes and cytoplasm of normal and tumor tissues and expressed in human lung, bronchus, nasopharynx, placenta, and lymphoid tissues (20, 21). In lung or respiratory tissues, activation of ADAM17 may contribute to the shedding of the collectrin-like part of ACE2, leading to the formation of soluble ACE2 (sACE2) (22, 23) and the development of inflammatory response (24). Furthermore, in distinct cells from the lung, ADAM17 expression is relatively high in pneumocytes and endothelial cells (20), suggesting that ADAM17 may be participating in the cleavage and shedding of key proteins in lung tissues. Activation of ADAM17 promotes the release of soluble fms-like tyrosine kinase 1 (sFlt1) in the placenta and induces preeclampsia (25). ADAM17 also induces T-cell activation in lymphoid tissues through the promotion of L-selectin hydrolysis and shedding (26). ADAM17 is lowly expressed in NK cells and its activation by IL-15 obstructs the proliferation of NK cells (19). Among multiple immune cells, ADAM17 is relatively high expressed in granulocytes and monocytes (20). ADAM17 mediates IL-6R shedding from neutrophils and induces apoptosis (27), which may be associated with a pro-inflammatory response mediated by the sIL-6R/IL-6 trans-signaling pathway (28).

## Structure of ADAM17

**Figure 1:**
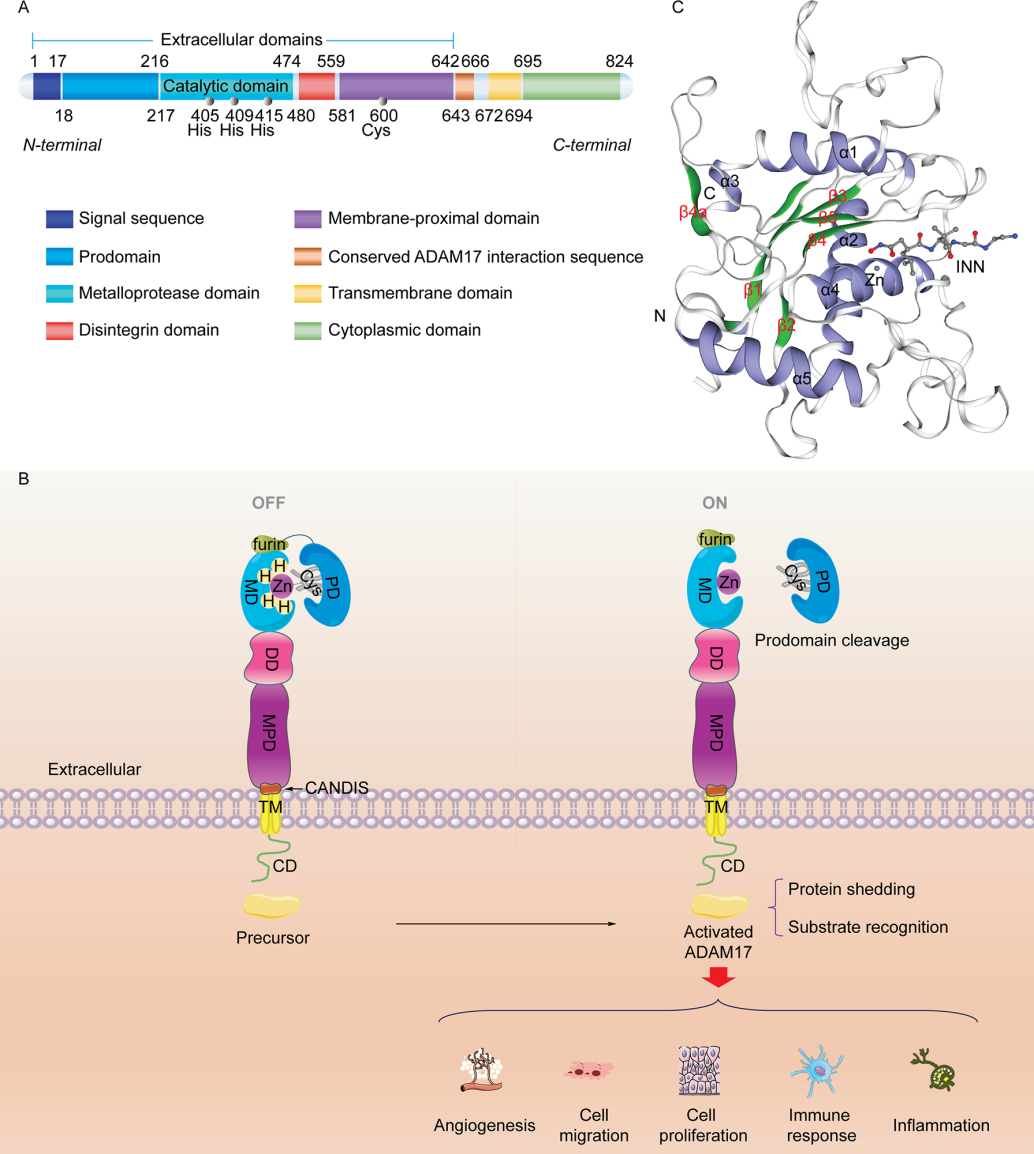
Molecular structure of the ADAM17 protein. **(A)** Sequence and structure of ADAM17. ADAM17 protein mainly comprises five extracellular domains, a transmembrane domain, and a cytoplasmic domain. **(B)** The classic cysteine-switch mechanism. The conserved cysteine switch is located in the prodomain. It coordinates with Zn^2+^ at the catalytic site of the metalloproteinase domain to produce an inactivated enzyme (ADAM17 precursor). Once its prodomain is cleaved, the adjacent furin site (RVKR sequence) is responsible for catalyzing the separation of zinc from cysteine, ultimately leading to ADAM17 activation. **(C)** The 3D catalytic structure of ADAM17 (PDB CODE: 1BKC) (31) with a hydroxamic acid-based inhibitor INN and Zn^2+^ shows N-terminal domains, α-helix (blue), β-sheet (green), and C-terminal domains. INN stands for N-{(2R)-2-[2-(hydroxyamino)-2-oxoethyl]-4-methylpentanoyl}-3-methyl-L-valyl-N-(2-aminoethyl)-L-alaninamide with the chemical structure of C19H37N5O5. INN, also known as TAPI-2, is an analogue of TAPI-1. This 3D image was made with the SWISS-MODEL Expasy.

ADAM17 is a member of the adamalysins subfamily of metzincin metalloproteinases consisting of 824 amino acids with zinc-dependent catalytic activities (29). The human ADAM17 protein sequence contains an N-terminal signal sequence (SS), a prodomain (PD), a catalytic metalloprotease domain (MD), a disintegrin domain (DD), a membrane-proximal protein domain (MPD), a conserved ADAM17 interaction sequence (CANDIS), a transmembrane domain (TM), and a C-terminal cytoplasmic domain (CD), which are located at amino acid residues 1-17, 18-216, 217-474, 480-559, 581-642, 643-666, 672-694, and 695-824, respectively (7, 30) (**Figure 1A**). Among them, the first five protein sequences that make up its extracellular domain may be involved in regulating multiple biological functions, including angiogenesis, cell migration, cell proliferation, inflammation, and immune responses. SS transfers the newly synthesized ADAM17 protein (110 kDa) to endoplasmic reticulum and Golgi apparatus (32). The PD obstructs the catalytic activity of metalloproteinases based on the cysteine-switch mechanism (33) (**Figure 1B**). During activation, furin, PC7 and PC5B pro-protein convertases are able to remove the prodomain of ADAM17 and induce production of the matured protein (80 kDa) (34). The cysteine-switch mechanism is not essential for the maintenance of inactivated ADAM17, which may be due to the presence of subdomains in the amino-terminal region of the prodomain (35). The MD serves as the main catalytic region of ADAM17 that contains a zinc-dependent HexGH-XXGXXHD motif (36). Amino acid residues His^405^, His^409^ and His^415^ located in this motif bind to zinc ions and determine the activity of the ADAM17 enzyme (31). The curved “Met turn” structure consisting of amino acid residues Tyr^433^, Val^434^, Met^435^, Tyr^436^, also known as 1,4-β-turn, is prone to ADAM17 cleavage and its mutations (37, 38). The DD can impair multiple functions of integrins, thereby affecting cell-cell/extracellular matrix interactions (20). In contrast to other members of ADAMs family, ADAM17 shows disulfide bonds in the MD, but its DD lacks typical calcium binding sites (39, 40). ADAM17 MPD plays crucial roles in substrate recognition and protein shedding. Due to the dimerization of ADAM17 and its substrate specificity, cysteine-rich and epidermal growth factor (EGF)-like domains are considered important components of MPD (41, 42). The hypervariable region of the former contributes to substrate recognition and shedding of extra-substrate domains and the latter affects the protein regulation of ADAM17 activity (43, 44). However, the existence of EGF-like domain remains controversial (45). In addition, the positively charged motif (Arg^625^-Lys^628^) in MPD binds to phosphatidylserine in the outer membrane, affects the conformation of ADAM17, and induces its activation (46). TM and CD mainly regulate the response of exocytodomain signaling molecule-related events (7, 38, 47), which may be attributed to the functional assembly of the Src SH3-binding motif (20). The CANDIS domain lies between MPD and TM, consisting of amino acid residues 643-666 (48), which binds to the type I transmembrane protein IL-6R but not the type II transmembrane protein TNF-α (49). As shown in **Figure 1C**, the visualized crystal structure of the catalytic domain of ADAM17 has five α-helices and five highly distorted β-sheet structures. The N-terminus binds to β1 and β3 sites, and the C-terminus binds to the α5 sites (31, 50). ADAM17 has shallower hydrophobic S1’ and very deep hydrophobic S3’ pockets linked by water channels, which facilitate the binding of the hydroxamic acid-based inhibitor TAPI-1 (also called an ADAM17 inhibitor) to the isobutyl side chain S1’ pocket and its other long chain to the S3’ pocket (31, 45). The structure and function of MD and MPD catalyzed by ADAM17 have been studied extensively, but the crystal structure and exact function of the remaining domains are still unclear.

## Regulatory roles of ADAM17

### ADAM17 regulates post-translational modification

**Figure 2:**
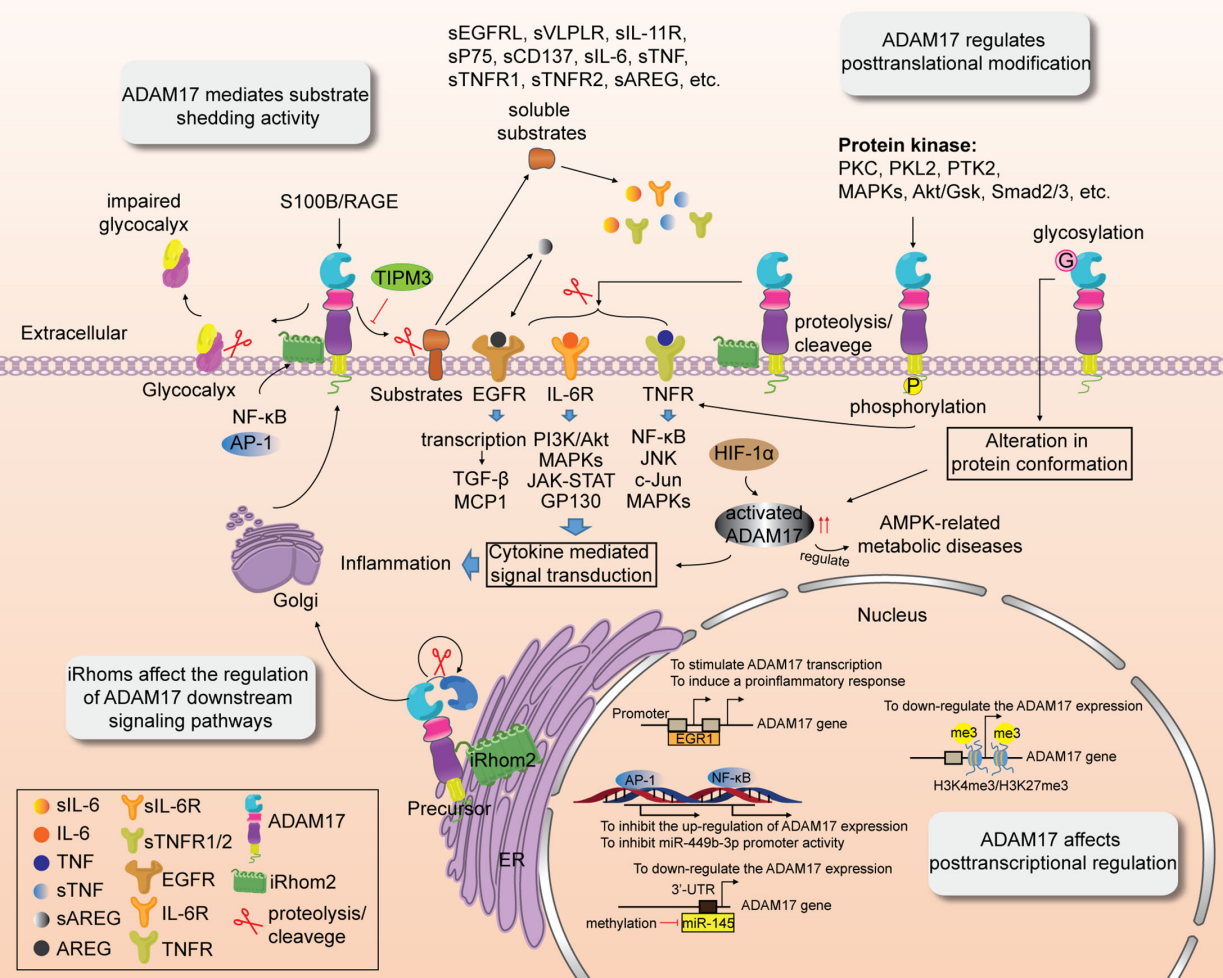
Multiple regulatory roles of ADAM17. ADAM17 activity is affected by transcriptional regulation, and post-transcriptional and post-translational modification. ADAM17 activity is also associated with substrate shedding. iRhoms affect the shedding of ADAM17 and regulation of its downstream signaling pathways.

Post-translational modification of precursor proteins includes proteolysis, phosphorylation, glycosylation, methylation and acetylation (51). It can regulate the hydrolysis and cleavage of proteins, affect their activities, localization and interaction with other cellular molecules. As an irreversible post-translational modification, proteolysis/cleavage of transmembrane proteins is responsible for activating multiple cytokine-mediated signal transduction pathways. ADAM17 was first identified as the TNF-α converting enzyme, and its transmembrane proteolysis is related to inflammation (52) and immune regulation (26). TNF consists of TNF-α and TNF-β, to be secreted by macrophages and/or T lymphocytes (53, 54). TNF-α interacts with its receptors TNFR1 and TNFR2. TNFR1 is widely expressed in various human cells and is involved in cell survival and cellular damage (55, 56). The death domain of TNFR1 is occupied by the silencer of death domains (SODD) which blocks the binding of TRADD to TNFR1 and suppresses the TNFR1 signaling pathway (57). The binding of TNF-α and TNFR1 enables the shedding of SODD from the death domain of TNFR1 and leads to the formation of the TNFR1-TRADD-RIP1-TRAF2 complex, thus promoting cell survival (57). In addition, TNFR1 is also internalized by the clathrin protein, which subsequently triggers the assembly of intracellular death-inducing signaling complex and activation of caspase8, leading to apoptosis or necrosis (57, 58). TNFR2 is mainly distributed in immune cells and plays a role in regulating the function of the immune system (59). Numerous studies have shown that furin endopeptidase close to the prodomain can remove the NH2-terminus of ADAM17 by proteolysis/protein cleavage (60), thereby activating it and inducing shedding of pro-TNF-α, TNFR1, and TNFR2 and subsequent pro-inflammatory response. Besides, ADAM17 triggers the hydrolysis and release of more than 90 substrate proteins. These have been further discussed in “ADAM17 Mediates Substrate Shedding Activity” section. Phosphorylation of ADAM17’s cytoplasmic tail is another post-translational modification. ADAM17 is often hyperphosphorylated in patients with emphysema (61). As shown in **Figure 2**, ADAM17 can be phosphorylated by various protein kinases, such as PKC (3), PKL2 (3), PTK2 (18), MAPKs (3, 62), Akt/GSK (63), and Smad2/3 (64). Recent studies have shown that the extracellular domain of ADAM17 with a homodimer structure can bind tightly to selective ADAM17 inhibitors, while serine 819 (Ser^819^) and threonine 735 (Thr^735^) in the cytoplasmic tail release selective ADAM17 inhibitors, which activate ADAM17 by inhibiting its phosphorylation-induced dimerization (65, 66). In addition, ADAM17 phosphorylation further promotes the shedding of TNF-α and two TNF receptors (32). Short term pro-TNF-α shedding by ADAM17 substrate does not depend on rapid phosphorylation of pro-TNF-α or the cytoplasmic tail of ADAM17 and it is mainly regulated by serine/threonine kinases (67). It has been reported that serine phosphorylation of ADAM17 substrate NRG1-ICD can restrain its cleavage of these post-translational modified substrates to some extent (68). Glycosylation of ADAM17 plays an important role in regulating enzyme activity or binding to substrates (69). ADAM17 glycosylation is significantly different between mammalian and insect cells (69). The glycosylation of ADAM17 cannot be detected in CRIB-1 cells (70). ADAM17-mediated TNF-α shedding is associated with O-glycosylation in the extracellular proximal membrane region (71). O-glycosylation at Ser41, however, prevented ADAM17-dependent cleavage of β1-AR (72). Glycosylation not only alters protein folding and conformation and affects ADAM17 activity, but also regulates receptor-mediated signal transduction (73, 74) and facilitates drug interventions targeting non-zinc-binding exosome sites of ADAM17 (69). Chen et al. found that zidovudine-based treatment inhibited the glycosylation of ADAM17 and the lysis of monocyte CD163 (75), indicating the important role of glycosylation in ADAM17 activity and disease progression.

### ADAM17 affects post-transcriptional regulation

In addition to post-translational modifications, ADAM17 also affects post-transcriptional regulation. ADAM17 is highly expressed or upregulated in cancer (76, 77) and other inflammation-related diseases, including kidney disease (78), sepsis (79), cicatrization (80), diabetic retinopathy (81), myocardial fibrosis (82), aortic dissection (83), arthritis (84) and atherosclerosis (7). The guanine-cytosine (G-C) sequences in the promoter region of ADAM17 are capable of binding specifically to many transcription factors (85–87). The gain- or loss-of-function of ADAM17 is attributed to the regulation of the following transcription factors, such as NF-κB (77, 88, 89), AP-1 (77, 88), SP1 (85), HIF-1α (82, 83), C/EBP-β (76), EGR1 (79), Sim1 (90), RUNX2 (91). For instance, inflammatory induction of inactive rhomboid protein 2 (iRhom2) stimulated by TNF and IFN-γ drives the activation and upregulation of ADAM17 expression and subsequent shedding of cell-surface molecules (77, 88, 89), which is blocked by NF-κB and AP-1 (77, 88, 89). However, ADAM17 can negatively regulate miR-449b-3p expression and its promoter activity *via* activating NF-κB transcription. MiR-449b-3p is a downstream target of ADAM17 and has a binding site of NF-κB in its promoter (77). He et al. found that EGR1 is bound to the ADAM17-172A>G (rs12692386) promoter region with affinity, leading to upregulation of ADAM17 promoter activity and transcription (79). However, the loss of EGR1 function prevents ADAM17 expression and induces a pro-inflammatory response. HIF-1α is an upstream target of ADAM17, and the transcriptional activation of HIF-1α promotes the upregulation of ADAM17 expression (82, 83). The latter regulates AMPK metabolism-related diseases through the adrenergic receptor (ADRA1A) (82). In addition, miR-145 downregulates ADAM17 expression by binding to the 3’-UTR of ADAM17, which leads to activation of the ADAM17-EGFR-Akt-C/EBP-β feedback loop and induction of tumor invasion (76). Epigenetic regulation of histone post-transcriptional modifications also plays a pivotal role in the post-transcriptional regulation of ADAM17. Recruitment/deletion of histone H3K4me3/H3K27me3 at the ADAM17 gene promoter downregulates ADAM17 expression (92), suggesting that dynamic chromatin modifications at this site lead to inflammatory responses.

### ADAM17 mediates substrate shedding activity

Due to the shedding activity, ectodomains of many transmembrane proteins are hydrolyzed and released by ADAMs metalloproteinases. Studies over the past five years revealed that ADAM17 has more than 90 substrates (7, 32) with distinct functions (**Table 1**), which are involved in various cellular processes, including cell adhesion, migration, development, inflammation, immune response, tumorigenesis, signal transduction. The cleavage and release of substrates (inflammatory cytokines, growth factors, receptors, adhesion molecules, and others) for ADAM17 may result in different functions of substrate proteins. Some substrate proteins, such as glycocalyx (104), TNFR (173, 178), and JAM-A/FIIR (156), are shed by ADAM17 in the form of active molecules. Glycocalyx is a polysaccharide protein complex that covers the aperture membrane surface of vascular endothelial cells and regulates the homeostasis of the cytoplasmic membrane through proteoglycan-glycoprotein attachment to endothelial cells. Recent studies have shown that activation of S100B/RAGE signaling by traumatic brain injury contributes significantly to ADAM17-mediated endothelial calyx shedding, which aggravates blood-brain barrier dysfunction and increased vascular permeability (104). The sheddase activity of ADAM17 drives scramblase-dependent phosphatidylserine (PS) exposure to the membrane surface, allowing the substrate to be cleaved and shed at the membrane surface (178, 179). The inability of ADAM17 to interact directly with PS may be due to the ability of the ortho-phosphorylserine form of PS to competitively inhibit the shedding of ADAM17 substrates (179). ANO6 facilitates the regulation of phosphatidylserine on the plasma membrane due to its scramblase activity. Veit M et al. found that downregulation of ANO6 expression by RNA interference significantly reduced the cleavage and release of TNFR1 by ADAM17 in HUVECs (178) and that free TNFR1 promotes TNF-induced cell necrosis (173). ADAM17-mediated JAM-A/FIIR shedding is responsible for aging-related abnormal endothelial remodeling (156). However, other substrates, like EGFR ligands (17, 97, 180), E-cadherin (124), VLDLR (4), IL-11R (5), CD137 (94), P75 (11), GPIBα (6), HPP1 (119), and NRG1 (10) are precursor proteins or fusion proteins that can yield active components or soluble active receptors only after cleavage and release by ADAM17 (**Figure 2**). Evidence suggests that ADAM17 promotes tumor-associated macrophage polarization and angiotensin II-mediated pro-growth and pro-migration signals by shedding EGFR ligands, including heparin-binding EGF-like growth factor (HB-EGF) and AREG (members of the EGF family), from the cell membrane (17, 32). E-cadherin is a key substrate for ADAM17, which is conducive to epigenetic regulation, endocytosis and efflux of cells by cleaving and shedding E-cadherin. Once ADAM17 binds to CD82, ADAM17 metalloproteinase activity is inhibited, leading to a reduction in E-cadherin cleavage products (124). IL-11 is a member of the IL-6 family that binds to IL-11R and forms a complex with CP130 to mediate anti-inflammatory signal transduction. On the other hand, IL-11R is hydrolyzed to soluble IL-11R (sIL-11R) *via* ADAM17 overexpression, mediating IL-11 trans-signaling pathway (5), which confers pro-inflammatory cytokine activity. Similarly, the bidirectional regulation of CD137/CD137L-mediated cellular responses has been implicated in the development of tumors and autoimmunity. The shedding protease ADAM17 triggers the production of soluble CD137 (sCD137), a spliceosome of CD137, which subsequently enhances T cell proliferation, whereas inhibition of ADAM17 activity intercepts the sCD137 production (94). VLDLR, an apolipoprotein receptor, plays an important role in foam cell formation, plasma triglyceride metabolism and inflammation. Its soluble ectodomain-mediated anti-inflammatory effect is related to the activation of the Wnt signaling pathway. ADAM17 induces the release of soluble VLDLR (sVLDLR), which inhibits the Wnt pathway and leads to macular degeneration in eye tissue, whereas the shedding of sVLDLR is blocked by selective ADAM17 inhibitors (4). Carrido et al. revealed that tumor formation mechanisms were probably caused by ADAM17-mediated cleavage of the P75 ectodomain (11). In addition, the increased ectodomain cleavage of other ADAM17 substrates (GPIBα and HPP1) may be required for immune platelet clearance and tumor suppression (119, 181). However, in another study related to oncogenic KRAS, KRAS mutations triggered enhanced ADAM17-mediated NPG1 shedding of the SLC3A2-NPG1 fusion protein, which in turn promoted tumor cell growth (10). Collectively, the pro-inflammatory and anti-inflammatory effects induced by ADAM17 substrate shedding may be related to distinct regulatory effects and functions of the substrates.

**Table 1:** Updated ADAM17 substrates (7, 32).

Cytokines	Growth factors	Receptors	Adhesion molecules	Others
CSF1 (93)	AREG (17)	4-1BB/CD137 (94)	ALCAM/CD166 (95, 96)	EGFRL (97)
CX3CL1 (98)	Epigen (99)	ACE2 (100, 101)	CD44 (102, 103)	Glycocalyx (104)
FLT-3L (105)	Epiregulin (106)	APP (107)	CD62L/L-selectin (108)	Klotho (109)
INFγ (110)	HB-EGF (111)	CA IX (112)	Collagen XVII (113–115)	Pref1 (116, 117)
Jagged1 (118)	HPP1/TMEFF2/Tomoegulin2 (119)	CD163 (75)	Desmoglein2 (95, 120)	SEMA4D (121)
Kit-ligand l and 2 (122)	NRG1 (10)	CD30 (123)	E-cadherin (124)	VASN/Vasorin (125)
LAG-3 (126)	TGFα (127)	CD40 (128, 129)	EpCAM (130)	
MICA (131)		CD89/FcαR (132)	ICAM1 (133)	
MICB (134)		c-MET (38, 135)	L1-CAM (136)	
RANKL (7, 137)		EMMPRIN/CD147 (138)	NCAM (139)	
TNFα (140)		EPCR (141)	Nectin4 (142)	
TNFβ (143)		ErbB4 (96)	PTP-LAR (144)	
		GHR (145)	VCAM1 (146)	
		GPIbα (6)		
		GPV (147)		
		GPVI (148)		
		IGFR1 (138)		
		IGF2R (149)		
		IL-11R (5)		
		IL-1RII (150, 151)		
		IL-6R (152, 153)		
		Integrin β1 (154, 155)		
		JAM-A/FIIR (156)		
		KIM1 (157)		
		LeptinR (158)		
		LOX1 (159, 160)		
		LRP1 (46, 161)		
		MEGF10 (162)		
		MerTK (163)		
		Notch1 (164)		
		NPR (32, 165)		
		p55TNFαR1 (140)		
		P75 (11)		
		p75 TNFR (127)		
		Ptprz (166)		
		PTPRA/PTPα (167)		
		sVLDLR (4)		
		Syndecan-1 and -4 (168)		
		TGFβR1 (169)		
		TIL4 (170)		
		TIM-3 (171, 172)		
		TNFR1 (173)		
		Trop2 (174, 175)		
		VEGFR2 (176)		
		VPS10P (177)		

### ADAM17 participates in the regulation of its downstream signaling pathways

ADAM17 regulates signal transduction in many pathophysiological processes, including inflammation, immunity and tumor. The upregulation of ADAM17 expression leads to increased EGFR ligand release and polarization of the EGFR signaling, which is responsible for cell proliferation, invasion, and migration (182, 183). However, downregulation of the ADAM17 expression urges the opposite effect by suppressing the EGFR/ERK, EGFR/Akt/C/EBP-β or EGFR/ErbB signaling pathways (76, 184). ADAM17-mediated EGFR signaling increases the levels of TGF-β and accumulates extracellular matrix (185), implying the role of TGF-β in the regulation of multiple immune cells under pro-inflammatory conditions. Emerging evidence suggest that blocking ADAM17 expression effectively alleviates inflammatory responses, which may be relevant to the regulation of pro-inflammatory cytokines IL-1β, IL-6 and TNF-α (186, 187). However, the loss of ADAM17 function with gene mutations triggers the development of inflammatory diseases (48, 188). Based on the aforementioned discussion, we suggest that ADAM17’s critical role in various signaling pathways ensures its activity is strictly regulated.

iRhoms, lacking the catalytic motif GxS, are members of the rhomboid protein family with important biological functions (189). Recently, iRhoms have been identified as key regulators of ADAM17 activation. In different tissues, iRhoms appear to form proteolytic complexes with ADAM17 sheddase, but not other ADAMs (190), thus mediating ADAM17 cell membrane surface transport. iRhoms contribute to the activation of ADAM17-dependent shedding events and substrate recognition, while deletion of iRhoms hinders ADAM17 activation, suggesting that iRhoms are required for ADAM17 maturation (190). iRhoms contain two inactive homologs, iRhom1 and iRhom2, also known as RHBDF1 and RHBDF2, respectively. iRhom1 is barely expressed in inflammatory/immune cells and yet iRhom2 is highly expressed in these cells and is responsible for ADAM17 activation (89). iRhom2 deficiency inhibits ADAM17-dependent substrate release, including bidirectional regulators and TNFs (191, 192). In iRhom2-mutated macrophages, ADAM17 remains in endoplasmic reticulum (ER), and cannot be activated by lysis of its prodomain (193). The cytoplasmic domain of iRhom2 participates in the regulation of ADAM17-dependent shedding events (189). Shed ADAM17 triggers phosphorylation of the N-terminus of iRhom2 and promotes the separation of ADAM17 from the iRhom2/ADAM17 complex by recruiting 14-3-3 protein (194). Despite the loss of protease activity, iRhom1 and iRhom2 maintain critical non-protease activities in regulating EGF and TNF-α signaling pathways (41, 195). Upregulated expression of iRhom1 in ER may enhance proteasome activity *via* the PAC1/2 pathway rather than *via* EGF signaling. Mice with iRhom2 deficiency had severe immunodeficiency and could neither produce the main inflammatory cytokine, TNF, nor could they respond to lipopolysaccharide-induced inflammation and immune responses. Therefore, iRhoms play an integral role in ADAM17-mediated downstream signal regulation. Hence, targeting iRhoms and ADAM17 may provide new strategies for anti-inflammatory treatment.

## Immune regulation of ADAM17 in cancers

### Abnormal expression of ADAM17 in cancers

Due to the shedding activity, ADAM17 is closely related to the formation and development of distinct cancer types, including lung cancer, ovarian cancer, breast cancer, stomach cancer, colorectal cancer, bladder cancer, melanoma, cervical cancer, pancreatic cancer, etc.

#### ADAM17 in lung cancer

Lung cancer has the highest incidence and mortality rate in the world. ADAM17 is usually an oncogene and its upregulation is associated with the progression of lung cancer. In LUAD, KRAS mutation contributes to the phosphorylation of ADAM17 threonine *via* p38 MAPK, thereby driving ADAM17 to selectively promote its substrate IL-6R shedding and subsequent ERK1/2 MAPK-IL-6-mediated trans-signal transduction, leading to malignant progression of the cancer (152). Enhanced ADAM17 activity mediated by KRAS mutation also facilitates the shedding of S-N (SLC3A2-NRG1) fusion protein NRG1 and the release of soluble NRG1 (sNRG1), which contributes to the increase in ERBB2-ERBB3 heterocomplex receptors and the activation of the downstream PI3K-AKT-mTOR pathway, leading to the growth of lung cancer cells (10). In addition, iRhom2, as a key binding protein for ADAM17, further promotes KRAS-induced tumor cell growth by modulating the release of ERBB ligands (196). However, the efficacy of radiotherapy for non-small cell lung cancer was enhanced when blockade of ADAM17 function with the neutralizing antibody (197). These findings suggest that ADAM17 is a cancer-promoting gene and a potential target for anti-lung cancer therapies.

#### ADAM17 in ovarian cancer

Fabbi et al. found that ADAM17 is significantly upregulated in ovarian cancer, and its high-expression is associated with poor clinical prognosis in ovarian cancer patients (198). High levels of ADAM17 in serum and ascites fluid of patients with ovarian cancer may be used as a hematologic tumor marker for the detection of ovarian cancer (199). ADAM17 promotes the malignant progression of ovarian cancer and causes chemo-resistance by mediating ADAM17-dependent shedding of AREG, HB-EGF, IL-6Rα, TNF, TNFR1-α, TGFα and activating the EGFR signaling pathway (198, 200). Deletion of ADAM17 or treatment with selective ADAM17 inhibitor GW280264X is capable of declining substrate cleavage/release and promoting chemo-sensitization (198, 201). ADAM17-induced P75 cleavage may also be responsible for ovarian cancer-promoting activities (11).

#### ADAM17 in breast cancer

ADAM17 functions as one of the highly expressed genes in breast cancer that plays an important role in the development of breast cancer. ADAM17 promotes cleavage of PD-L1 on the surface of breast cancer cells (12), regulates the interaction between PD-L1 and PD-1 (12), and may contribute to immune escape of triple-negative breast cancer cells (12, 202). ADAM17 can mediate the release of sTNFR1 and sTNFR2, which inhibit the secretion of metastasis-promoting chemokines (CXCL8, CCL5, CXCL) and induce anti-metastasis effects in triple-negative breast cancer cells (203). An earlier study indicated that breast cancer-associated fibroblasts stimulated breast cancer cell proliferation through ADAM17-mediated cleavage of TGF-α (204). Interestingly, ADAM17 is also present in platelets and is involved in tumor immune escape. It was found that downregulation of ADAM17 in activated platelets from breast cancer patients was associated with tumor metastasis and clinical stage of breast cancer (14). D8P1C1, an anti-ADAM17 monoclonal antibody, remarkably inhibited tumor growth in triple-negative breast cancer mouse models (205). Similar results were reported in another published paper (13). In summary, the critical role of ADAM17 in breast cancer makes it a potential target for breast cancer therapy.

#### ADAM17 in gastric cancer

ADAM17 is probably associated with aggressive metastasis and poor prognosis of gastric cancer. A meta-analysis associated with gastric cancer indicated that ADAM17 might be a tumor marker for poor prognosis in gastric cancer, and high expression of ADAM17 is associated with lymph node metastasis and clinical staging of lymph node metastasis in gastric cancer (15). ADAM17 promotes gastric cancer cell metastasis by activating the Notch-Wnt signaling pathway (206). Epithelial-mesenchymal transition (EMT) is a transformation of cell morphology that occurs in the development of tumors, including gastric cancer. It was reported that ADAM17 promotes EMT in gastric cancer cells (33, 64). The mechanism of ADAM17 in gastric cancer may be through TGF-β/p-Smad2/3-mediated EMT activation (207, 208).

#### ADAM17 in other cancers

ADAM17 is also highly expressed in cervical cancer, liver cancer, colorectal cancer and bladder cancer. ADAM17-modified bone marrow mesenchymal stem cells may stimulate the malignant growth of drug-resistant cervical cancer cells by activating the EGFR/PI3K/Akt pathway (16). ADAM17 is thought to cleave the Notch receptor and inactivate Notch signaling, thereby impeding the GPR50/ADAM17/Notch axis-mediated development of liver cancer (85). ADAM17 can interact with cellular integrin α5β1 to promote the binding and uptake of exosomes derived from colorectal cancer (209). Newly formed exosomes are associated with the malignant phenotype of tumors. In addition, ADAM17 also promotes STAT3 activation by induction of EGFR/IL-6 transduction signaling pathways, which ultimately lead to tumor progression; inhibition of the ADAM17/IL-6/STAT3 signaling axis significantly attenuated the growth of colon cancer cells (210). The ADAM17/EGFR/AKT/GSK3β axis plays a key role in regulating melanoma cell proliferation, migration, and cell sensitivity to chemotherapeutic drugs (211). ADAM17 is also involved in immune-related autocrine and paracrine regulation (40). However, knockdown of ADAM17 or treatment with anti-ADAM17 antibody MEDI3622 resulted in regression of pancreatic tumors, accompanied by down-regulated EGFR/STAT3 signaling, increased cytotoxic T cells, and decreased granulocyte-like medullary inhibitory cells in mouse models of pancreatic cancer (212).

### Regulation of macrophages by ADAM17

Tumor microenvironment (TME) refers to a complex environment closely related to tumorigenesis, tumor growth and its metastasis, which is composed of a variety of cells (including macrophages, fibroblasts, lymphocytes, endothelial cells, etc.), extracellular matrix, and multiple signaling molecules (cytokines, growth factors, chemokines, hormones, etc.) (213). Autocrine and paracrine are conducive to the activation of multiple signaling pathways in tumor cells and non-tumor cells (e.g., macrophages, lymphocytes, endothelial cells) (214–216). In this way, the dynamic interaction between tumor cells and their surrounding matrix triggers tumor cell proliferation, immune evasion, distal metastasis, and drug resistance, and angiogenesis as well (217, 218).

Tumor-associated macrophage (TAM) is derived from mature monocyte in peripheral blood. Monocytes are recruited to TME through chemokines and cytokines secreted by tumor cells and become TAMs. TAMs are the most abundant immune cells in the TME and are closely relevant to tumor growth, invasion and metastasis (219). For one thing, macrophages serves as an important component of tumor stromal cells that are able to gather around blood vessels and promote angiogenesis, tumor invasion and metastasis (220, 221). For another thing, it also has the ability to remove tumor cells and reshape the TME (222). Due to the influence of cytokines in the TME, TAMs can be divided into two distinct polarized forms, M1 and M2 macrophages. The former is responsible for killing tumor cells; the latter is able to promote tumor growth (223). Macrophage M1/M2 polarization is adjustable and reversible. Increased M2-polarized TAMs are often associated with cytokines and growth factors, e.g., IL-4 (224), IL-10 (225), CSF-1 (226), TGF-β (227) secreted by tumor cells or Th2 cells in the TME, indicating a poor prognosis for tumor patients.

**Figure 3:**
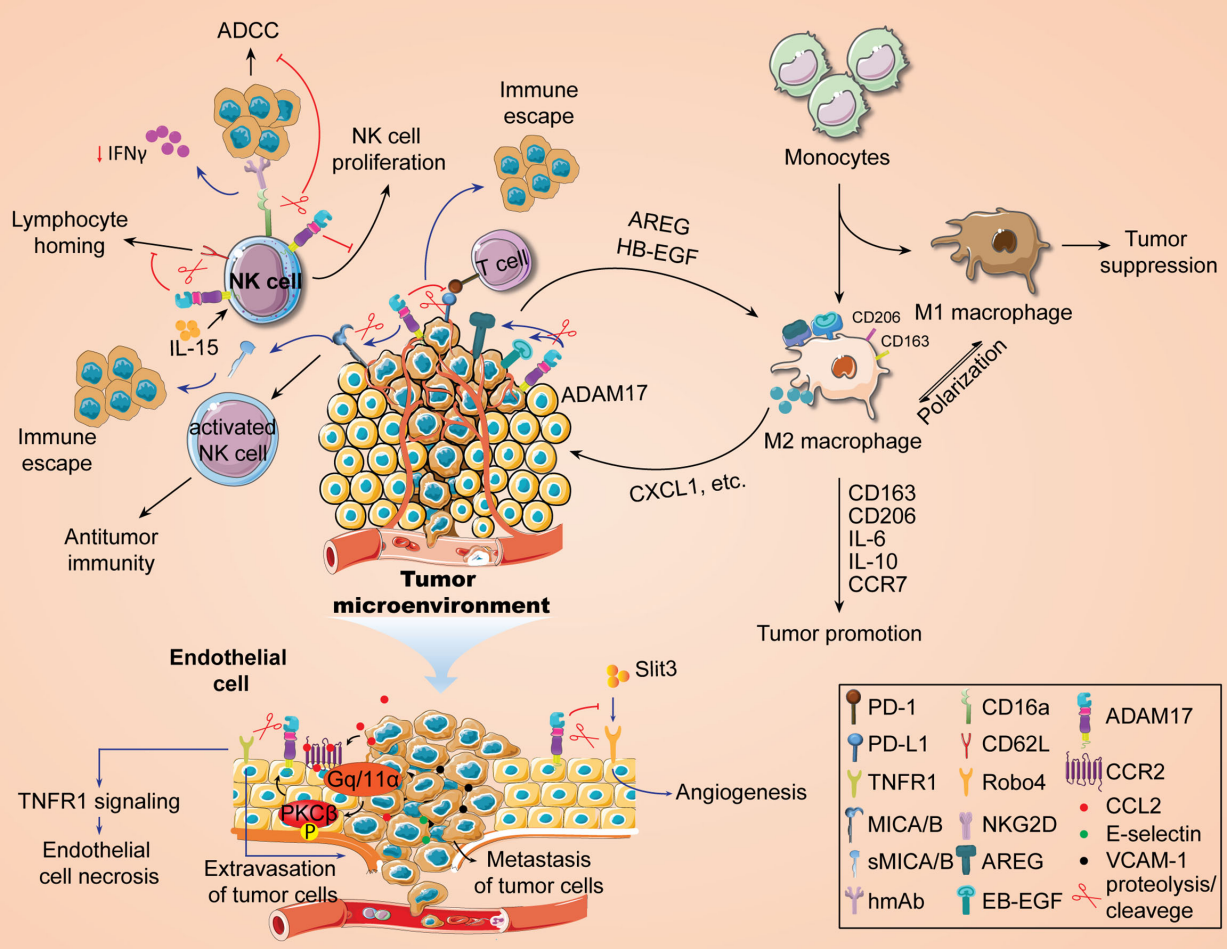
Immunomodulatory role of ADAM17 in tumor development.

Metalloproteinase ADAM17 can shed distinct signaling proteins on the cell surface, making it a mediator for intercellular signal transduction (7, 20). Our previous study showed that the expression of ADAM17 was associated with infiltration of multiple immune cells, including macrophages (20), in TCGA pan-cancer samples and yet the specific regulatory mechanism of ADAM17 is unknown. Recently, Gnosa et al. have revealed the positive roles of ADAM17 in regulating the polarization of TAMs (17). By using bioinformatics analysis based on the TCGA dataset and immunohistochemical analysis from triple-negative breast cancer cohort, the authors first confirmed that highly expressed ADAM17 in tumors is positively correlated with tumorigenic macrophage markers CD163 or CD206. Deletion of ADAM17 gene inhibited tumor growth, increased the survival in tumor-bearing mouse models, and resulted in a significant decrease in CD163+ cell population. In a co-cultured mouse bone marrow-derived macrophages with ADAM17-WT or ADAM17-KO tumor cells, knockdown of ADAM17 significantly diminished the expression of CD163 or CD206, IL-6, IL-10, and CCR7 in bone marrow-derived polarized macrophages, suggesting an important role of ADAM17 in tumorigenic macrophages. Furthermore, the authors used cellular co-culture and zebrafish embryo propagation models to demonstrate that tumor cells, in an ADAM17-dependent manner, drive macrophage polarization into a tumor-promoting phenotype and accelerate tumor cell invasion. Based on the sheddase activity of ADAM17 (38), this macrophage polarization is regulated by ADAM17-mediated shedding of EGFR ligands (HB-EGF, AREG). Actually, the mechanism of macrophage polarization driven by tumor cells has been reported in many previous works. For instance, the EGFR/PI3K/AKT/mTOR axis plays an important role in promoting TAM M2 polarization by secreting EGF from colon cancer cells (228). Pancreatic cancer triggers the polarization of TAM M2 by secreting REG4 through the EGFR/AKT/CREB pathway (229). These findings further indicate that EGFR ligand shedding mediated by ADAM17 may be beneficial to activating the EGFR signaling pathway and inducing the polarization of tumor-promoting TAMs. Finally, they further demonstrated the promoting effect of macrophage-derived CXCL1 secretion on tumor cell invasion by RNA-seq analysis of transcriptome data from co-cultured macrophages. Taken together, these findings suggest a critical role of the ADAM17-EGFR (HB-EGF/AREG) axis in the polarization of TAMs (**Figure 3**), which also provides a new strategy for the anti-tumor immunotherapy.

### Regulation of NK cells by ADAM17

Natural killer (NK) cells are important lymphocytes to fight against tumor escape or immune evasion. A large number of studies have shown that the activity and function of NK cells in peripheral blood of some cancer patients are significantly reduced (230), which may be conducive to the development of malignant tumors. In non-small cell lung cancer, the function of NK cells has been shown to be significantly impaired. Therefore, immunotherapy targeting NK cells has become a therapeutic concept for this type of cancer (231). One of the reasons for the anti-tumor immune activity of NK cells is attributed to the binding of its surface-activated receptor natural killer cell group 2D (NKG2D) to MHC class I chain-related protein A/B (MICA/B), an NKG2D ligand on the surface of tumor cells, thus activating NK cell function and enables NK cells to kill tumor cells (232). Studies have shown that inhibition of the ADAM9 activity significantly blocked MICA shedding and affected the immune killing effect of NK cells on tumor cells (233). ADAM17 is also a member of the metalloproteinase family that may have a similar function. Recently it was found that ADAM17 has the ability to hydrolyze MICA/B on the surface of tumor cells to generate soluble MICA/B (sMICA/B) (52), the latter of which alters the conformation of NKG2D on the surface of NK cells (234) and affects the recognition and binding of membranous MICA with NKG2D, thereby inhibiting NK activation signals and reducing the killing sensitivity of NK cells to tumor cells (235). Knockdown of ADAM17 prohibits MICA shedding and boosts MICA expression on the surface of hepatocellular carcinoma cells (131). In addition, hypoxia-induced shedding of MICA on the surface of pancreatic cancer cells enables tumor cells to evade NK cell immune killing (235). The function of MICA/B monoclonal antibodies is to inhibit MICA/B shedding by binding antibodies at key epitopes in the MICA/M proximal membrane domain, and its antitumor immunity activity is associated with NKG2D and CD16 Fc receptor activation (236). Inhibition of MICA/B shedding with monoclonal antibodies drives NK cell-mediated antitumor immunity (237), suggesting that the sMICA levels may be correlated with decreased NK cell function. Therefore, blocking ADAM17-mediated hydrolytic activity to inhibit MICA shedding may be one of the ways to improve NK cell killing of tumor cells (**Figure 3**).

The antitumor immune activity of NK cells is also related to the antibody-dependent cell-mediated cytotoxicity (ADCC) induced by CD16 Fc receptor (**Figure 3**). ADCC is a key cytolytic mechanism of NK cells. NK cells, on the one hand, interact with the Fc region of antibodies that recognize proteins on the surface of tumor cells through their IgG Fc receptors to target tumor antigens and produce cytotoxic effects. On the other hand, it also mediates adaptive immune responses. In human beings, IgG’s FcR family consists of six receptors, including FcγRI (CD64), FcγRIIa (CD32a), FcγRIIb (CD32b), FcγRIIc (CD32c), FcγRIIIa (CD16a), and FcγRIIIb (CD16b), of which CD16a is primarily responsible for triggering NK cell-mediated ADCC. Therefore, exploring the mechanism of CD16a contributes to the development of anti-tumor immunotherapy drugs that enhance ADCC activity. The metalloprotease ADAM17 has been reported to shed CD16a (238), leading to decreased ADCC activity and reduced IFN-γ production (239). However, Blocking CD16 shedding or avoiding cleavage prompted a stronger tumor cell killing by NK cells (240, 241) and increased IFN-γ production (242). Paradoxically, treatment with an ADAM17 inhibitor did not increase IFN-γ levels induced by stimulated NK cells (242). CD16a is a hot topic discussed in recent NK cell anti-tumor immunity, and more information about the role of ADAM17 in the regulation of CD16a in NK cells can be seen in some recent studies (238, 243).

In addition, IL-15, an immunomodulatory factor, also plays a key role in the development, homeostasis, activation and proliferation of NK cells (244). IL-15 can differentiate hematopoietic progenitor cells into CD56+ NK cells to induce pro-proliferative responses. In NOG-IL-15 Tg mice expressing transgenic human IL-15, there is a significant increase in transplanted NK cells from healthy subjects’ peripheral blood (19, 245). In different tumor-bearing animal models, IL-15 treatment contributes to tumor regression, reduction of tumor metastasis, and improvement of animal survival. Currently, the developed IL-15 mutant (IL-15N72D) or its stable soluble complex, ALT-803, has been shown to have similar functions as IL-15 and significantly improved the antitumor activity of anti-CD20 monoclonal antibody in NK cells and the immunotherapeutic efficacy of PD-1/PD/L1 monoclonal antibody (232). ADAM17 is present in various immune cells, including NK cells (20), which mediates lysis and shedding of cell surface receptors. CD62L/L-selectin is an immune cell homing receptor that regulates the migration of white blood cells to sites of inflammation. It was found that CD62L expression is increased in IL-15-stimulated NK cells (19). Expression of ADAM17 on NK cells promotes the downregulation of CD62L expression (242). Mishra et al. first indicated that ADAM17 reduced IL-15-stimulated NK cell proliferation with the participation of CD62L (19). The blockade of ADAM17 reversed this event. Overall, IL-15-mediated NK cell proliferation promotes an increase in CD62L levels, while prolonged activation of ADAM17 leads to CD62L shedding and impaired NK cell proliferation stimulated by IL-15 (**Figure 3**).

### Regulation of endothelial cells by ADAM17

Metastasis is a form of tumor progression. 90% of tumor-related deaths are caused by metastasis of tumor cells. The process includes: 1) the shedding of tumor cells from the primary tumor; 2) intravasation; 3) survival in the blood circulation; 4) extravasation of blood vessels and metastases. The interaction between endothelial cells and tumor cells is an important step in tumor metastasis. Tumor cell-endothelial cell tight contacts promote tumor cell adhesion to the vascular wall through justacrine or paracrine signaling (246). As shown in **Figure 3**, endothelial cells secrete a series of adherent molecules, such as E-selectin, VCAM-1, etc., to increase the adhesion of tumor cells with endothelial cells, and further promote tumor metastasis. The mechanism of tumor metastasis may be related to EMT, angiogenesis, tumor stem cell characteristics, and the increase of circulating tumor cells. ADAM17 is widely present in endothelial cells and is positively correlated with immune infiltration levels of endothelial cells in multiple cancer species (20, 88, 173). It is speculated that endothelial ADAM17 may help tumor metastasis. Recent emerging evidence supports this speculation (173). Julia et al. also confirmed that endothelial ADAM17 is required for endothelial necrosis, tumor cell extravasation and metastasis (247). ADAM17-dependent death receptor TNFR1 ectodomain shedding promotes endothelial cell necrosis and tumor cell extravasation (173, 247). In addition, CCL2 secreted by tumor cells and macrophages promotes PKCβ activation by binding to endothelial CCR2, which further leads to ADAM17 activation (247). ADAM17 appears to be closely associated with pathological angiogenesis (138). In ADAM17flox/flox/Tie2-Cre mice, loss of endothelial ADAM17 inhibits chord formation and impedes ectopic injected tumor growth (138). In endothelial cells, soluble Robo4 (sRobo4) is shed and released by ADAM1, which subsequently inhibits SLIT3-induced angiogenesis (248). Meanwhile, SLIT3 obstructs Robo4 shedding and enhances its signal transduction (248). ADAM17 may disrupt the barrier effect of vascular endothelial cells by affecting their attachment and tight junctions (249). Beyond vascular endothelial cells, ADAM17 is also important in lymphatic endothelial cell-induced tumor migration and metastasis. Sun et al. indicated that ADAM17 activation by MAPK14/T180 promoted the secretion of soluble CX3CL1, which further led to malignant metastasis of liver cancer cells (18). In addition, Macrophage M2 polarization is also associated with ADAM17-dependent CX3CL1 secretion (18). As a critical binding protein for ADAM17, iRhom1 has been found to promote independent regulation of ADAM17 under physiological shear stress (88). However, there is no report yet on the regulation of ADAM17 by iRhom1 in endothelial cells and its effect on tumor malignant progression, which may be an interesting topic.

## ADAM17 inhibitors

**Figure 4:**
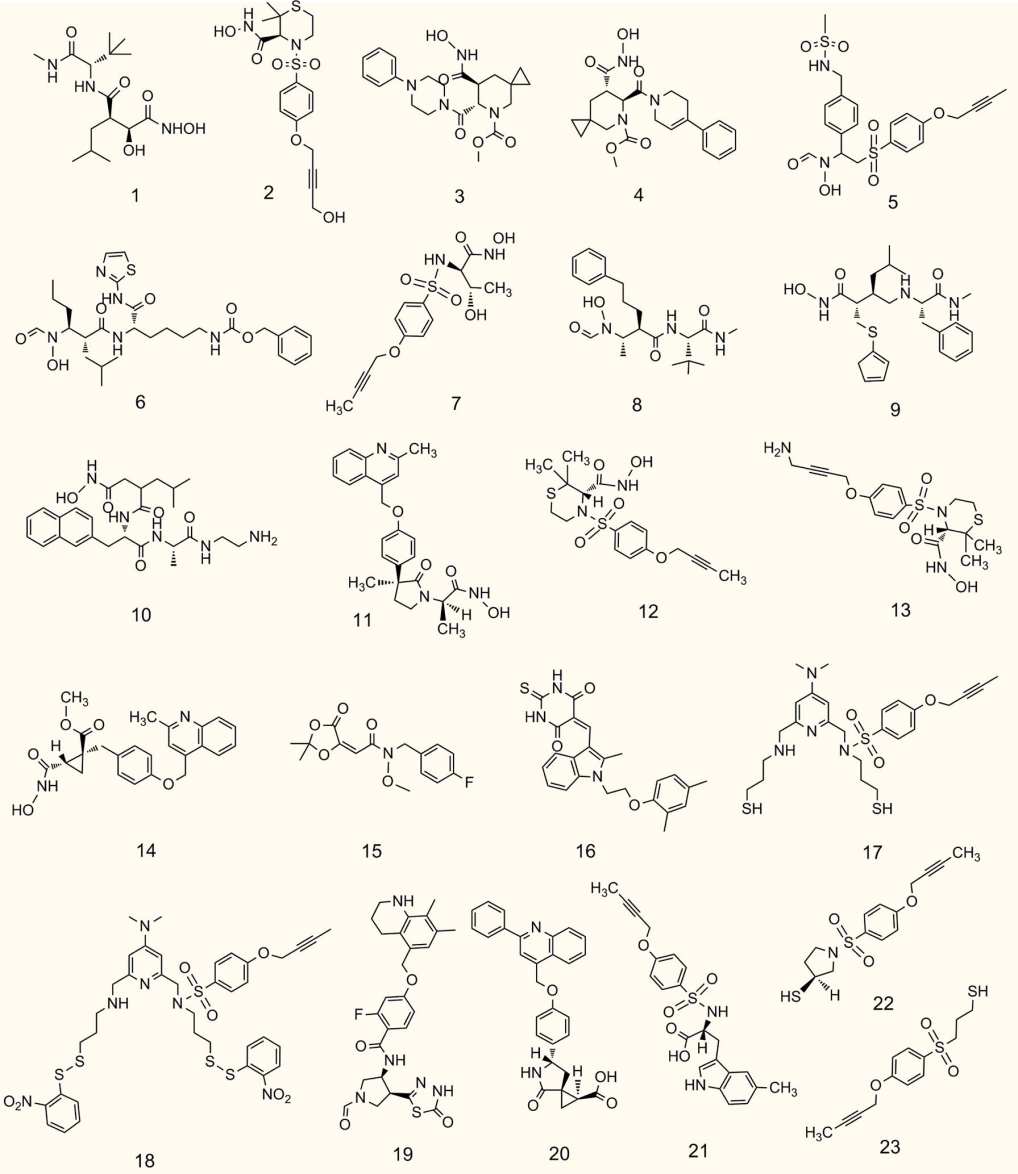
Chemical structures of representative small molecule ADAM17 inhibitors.

ADAM17 has over 90 substrates, some of which are mediators of cancer diseases, which implies that substrate based ADAM17 inhibitors have the potential to be used for the treatment of malignant tumors. In this section, we outline recent advances in potent and selective ADAM17 inhibitors containing hydroxamate and non-hydroxamate moieties, as well as anti-ADAM17 monoclonal antibodies (**Figure 4**, **Table 2**).

**Table 2:** Summary of the inhibitory activities of ADAM17 inhibitors.

Compound Number^a^	Chemical Name or Product Name	IC_50_ Value^b^	Reference
**Hydroxamate-based small-molecule compounds:**			
1	Marimastat	4.75 μM	(102)
2	Apratastat	144 ng/mL (*in vitro*); 81.7ng/mL (*ex vivo*)	(250)
3	Aderbasib/INCB7839	*N.D.*	(251)
4	INCB3619	14 nM	(252)
5	KP-457	10.6 nM	(253)
6	GW280264X	*N.D.*	(4, 201, 254)
7	PF-5480090/TMI-002	~1696.5 RFu/mg	(255)
8	GI254023X	541 μM	(256)
9	Batimastat	*N.D.*	(257)
10	TAPT-1	8.09 μM	(107)
11	(2R)-N-hydroxy-2-[(3S)-3-methyl-3-{4-[(2-methylquinolin-4-yl)methoxy]phenyl}-2-oxopyrrolidin-1-yl]propanamide	*N.D.*	(20)
12	(3S)-4-{[4-(but-2-ynyloxy)phenyl]sulfonyl}-N-hydroxy-2,2-dimethylthiomorpholine-3-carboxamide	*N.D.*	(20)
13	(3S)-4-{[4-(but-2-ynyloxy)phenyl]sulfonyl}-N-hydroxy-2,2-dimethylthiomorpholine-3-carboxamide	*N.D.*	(20)
14	Methyl (1R,2S)-2-(hydroxycarbamoyl)-1-{4-[(2-methylquinolin-4-yl)methoxy]benzyl}cyclopropanecarboxylate	*N.D.*	(20)
15	BMS-561392	0.20 nM	(258)
**Non-hydroxamate-based small-molecule compounds:**			
16	ZLDI-8	6.85 μM	(259)
17	SN-4	3.22 μM	(102)
18	SN-4(Nps)_2_	*N.D.*	(102)
19	JTP-96193	5.4 nM	(258)
20	(1S,3R,6S)-4-oxo-6-{4-[(2-phenylquinolin-4-yl)methoxy]phenyl}-5-azaspiro[2.4]heptane-1-carboxylic acid	*N.D.*	(20)
21	N-{[4-(but-2-yn-1-yloxy)phenyl]sulfonyl}-5-methyl-D-tryptophan	*N.D.*	(20)
22	(3S)-1-{[4-(but-2-yn-1-yloxy)phenyl]sulfonyl}pyrrolidine-3-thiol	*N.D.*	(20)
23	3-{[4-(but-2-yn-1-yloxy)phenyl]sulfonyl}propane-1-thiol	*N.D.*	(20)
**Anti-ADAM17 monoclonal antibodies:**			
	A300E	~0.7 μg/mL	(260)
	A9(B8)	0.22 nM (human); 0.25 nM (mouse)	(261)
	D1(A12)	4.7 nM	(262)
	MEDI3622	39 pmol/L (human); 132 pmol/L (mouse)	(263)

^a^See **Figure 4**; ^b^N.D. refers to not detected.

### Hydroxamate-based small-molecule inhibitors

The metalloproteinase domain of ADAM17 has a catalytic site containing a sequence of zinc-dependent amino acid residues that can bind to zinc ions to interfere with ADAM17 enzyme activity. The hydroxamate moiety is a common zinc-binding motif, and hydroxamate-based small-molecule inhibitors targeting the catalytic site may be an effective strategy against tumors. Marimastat and apratastst are the earliest synthesized hydroxamate-based inhibitors with limited selectivity. Marimastat inhibits the cleavage of TNF-α and CD44 and reduces the invasion of tumor cells with an IC_50_ of 4.75 μM (102). Shu et al. found that apratastst significantly inhibited TNF-α cleavage with IC_50_ of 81.7 ng/mL *ex vivo* and 144 ng/mL *in vitro*, respectively (250). INCB7839 is not ideal as a single agent, but it enhances the efficacy of trastuzumab in metastatic HER2-positive breast cancer. INCB7839 suppresses ADAM10/17-dependent EGFR ligand shedding and potentiates the antitumor effects of the recombinant peptidase PEPDG278D (251). Since January 2009, INCB7839 has been used in Phase I/II clinical trials alone or in combination with rituximab/trastuzumab + vinorelbine/trastuzumab + docetaxel for the treatment of diffuse large B cells non-hodgkin lymphoma gliomas, breast cancer or solid tumors (**Table 3**). In a subset of subjects, INCB7839 at a dose of 300 mg b.i.d. (Phase II) in combination with rituximab resulted in a range of serious side effects, including thromboembolism, pain, and infections (NCT02141451). However, other anticancer clinical trials associated with INCB7839 were terminated for some reason or were not conducted or not yet reported (NCT01254136; NCT00864175; NCT0429575; NCT00820560). INCB3619, an early hydroxamate-based inhibitor with the IC_50_ value of 14 nmol/L, significantly inhibits tumor cell survival by blocking the shedding of ErbB ligands (252). INCB3619 also enhances the sensitivity of gefitinib (264), cisplatin (252), and lapatinib (265), and acts synergistically with CD16 × 33 bispecific killer cell conjugates against acute myelogenous leukemia (266). KP457 increases the production of platelets derived from functional human induced pluripotent stem cells by inhibiting the exodomain shedding of platelet glycoprotein Iba (GPIba), with an IC_50_ value of 10.6 nmol/L for KP457 (253). GW280264X facilitates the anti-ovarian cancer effect of cisplatin (201) and restrains the development of lung adenocarcinoma cells (254). The IC_50_ value of PF-5480090/TMI-002 in MDA-MB-468 cells is approximately 1696.6 RFU/mg, which reduces the release of TGF-α and increases the cytotoxic effects of anti-EGFR/HER drugs (255). GI254023X is a selective inhibitor of ADAM10 and ADAM17, but its selectivity for ADAM10 is 100 times higher than that of ADAM17, with IC_50_ values of 5.3 μM and 541 μM for ADAM10 and ADAM17, respectively (256). The hydroxamate derivative batimastat inhibits ADAM17 shedding (267) and has prevented the progression of multiple tumors in clinical trials, particularly the formation of peritoneal carcinomas (268). TAPI-1 with IC_50_ value of 8.09 μM is capable of inhibiting matrix metalloproteinase and blocking the shedding of cytokine receptors (107). Recent studies have shown that TAPI-1 appreciably restrains ADAM17 activation during pseudomonas aeruginosa infection (269). Additionally, we previously retrieved four novel hydroxamate-based small molecule compounds 11-14 targeting ADAM17 from the DrugBank database, but no *in vitro* and *in vivo* experimental data were reported (20). BMS-561392 reduced ADAM17 activity with an IC_50_ of 0.2 nM. Overall, most hydroxamate-based inhibitors exhibit potent ADAM17 shedding activity and resist tumor progression. Compounds with the hydroxamate group, however, are usually poorly bioavailable and produce toxic hydroxylamine through metabolism, which somewhat limits the clinical use of these compounds (270).

**Table 3:** Currently approved clinical trials using ADAM17 inhibitors for tumor treatment.

Diseases	ADAM17 inhibitors	Phase for trial	Trial ID	First Posted date	Recruitment Status	Last Update Posted
Diffuse Large B Cell Non-Hodgkin Lymphoma	INCB7839 + Rituximab	Phase I/II	NCT02141451	May 19, 2014	Completed	Feb 19, 2020
Gliomas	INCB7839	Phase I	NCT04295759	Mar 4, 2020	Active, not recruiting	Aug 16, 2022
Breast Cancer	INCB007839 + Trastuzumab and Vinorelbine	Phase I/II	NCT01254136	Dec 6, 2010	Terminated	Jan 25, 2012
Breast Cancer	INCB007839 + trastuzumab and docetaxel	Phase I/II	NCT00864175	Mar 18, 2009	Terminated	Jan 18, 2018
Solid Tumors and Hematologic Malignancy	INCB007839	Phase I	NCT00820560	Jan 12, 2009	Completed	Jan17, 2018

(Source: the U.S. National Library of Medicine, https://clinicaltrials.gov/).

### Non-hydroxamate-based small-molecule inhibitors

To avoid side effects and toxicity caused by the hydroxamate group and to improve bioavailability, research on new ADAM17 inhibitors has been directed toward non-hydroxamate-based small-molecule compounds (46). By searching the literature published in the last five years, we have selected the following four new compounds for description. With computerized virtual screening, Lu et al. identified a non-hydroxamate-based inhibitor, called thioxodihydro pyrimidindione ZLDI-8, which reversed taxol resistance, displayed an IC_50_ value equal to 6.85 μM against ADAM17 (259), and inhibited metastasis of hepatocellular carcinoma (271). It also enhanced the antitumor effects of sorafenib and 5-fluorouracil (272, 273). Another non-hydroxamate-based inhibitor, SN-4 specifically impedes ADAM17-mediated cleavage of TNF-α and CD44 with a higher activity than malistamate and an IC_50_ value of 3.22 μM (102). SN-4(Nps)_2_, a prodrug of SN-4, can markedly enhance its bioavailability. A thiadiazolone derivative JTP-96193 showed 1800 times more selectivity toward ADAM17 over other matrix metalloproteinases with an IC_50_ value of 5.4 μM (258). Compounds 20-23 are novel non-hydroxamate-based small molecules targeting ADAM17 from the DrugBank database, whereas their ADAM17 inhibitory activity and potential mechanism remain to be further explored (20).

### Anti-ADAM17 monoclonal antibodies

The development of anti-ADAM17 monoclonal antibodies has accelerated the progress of innovative ADAM17 inhibitors. Anti-ADAM17 monoclonal antibodies include A300E, A9 (B8), D1 (A12), MEDI3622, etc. A300E is rapidly internalized by ADAM17-expressing cells (274), and its IC_50_ against ADAM17 is approximately 0.7 μg/mL (260). Trad et al. suggested that A300E plays a role in cancer cells by transporting a conjugated toxin to target cells (260). A9 (B8) cross-reacts with both human and mouse ADAM17, whereas D1 (A12) binds only to human ADAM17. D1 (A12) is bound to both the catalytic and non-catalytic domains of ADAM17. Yang et al. found that A9 (B8) conferred EGFR-TKI-mediated antitumor effects in NSCLC cells with IC_50_ values of 0.22 nM and 0.25 nM against human and murine ADAM17, respectively (261). Ye et al. revealed that A9 (B8) inhibited the shedding of ADAM17 substrate and contributed to the growth inhibition of pancreatic ductal adenocarcinoma *in vivo* and *in vitro* (275). D1 (A12) at 4.7 nM inhibits 50% TNF-α shedding and induces anti-ovarian cancer effects (262). Besides, D1 (A12) restrains the progression of head and neck squamous cell carcinoma by reducing HERS-transactivation induced by retarded hormone and even has therapeutic prospects for EGFR TKI-resistant head and neck squamous cell carcinoma (276). Another anti-ADAM17 monoclonal antibody, MEDI3622, inhibits tumor-dependent EGFR activity with IC_50_ values of 39 pmol/L and 132 pmol/L against human and murine ADAM17, respectively (263). In esophageal and colorectal tumors, the antitumor effect of MEDI3622 was superior to that of the EGFR/HER pathway inhibitor, suggesting that MEDI3622 inhibits tumor growth by partially modulating non-EGFR-mediated pathways (263). In addition, MEDI3622 enhances the release of antibody-bound tumor cells binding IFN-γ in NK cells by blocking CD16A shedding (239).

To date, there are no clinically available ADAM17 inhibitors. The high toxicity and low selectivity of existing ADAM17 inhibitors and the high structural homology between the catalytic domain of ADAM17 and other metalloproteases (e.g., ADAM10) have limited the development of selective ADAM17 inhibitors. However, the advent of small molecule compounds and anti-ADAM17 monoclonal antibodies targeting the non-catalytic domain of ADAM17 or the catalytic and non-catalytic domains of ADAM17 (44, 46, 277–279) further overcome these problems and improve bioavailability, which may provide a new strategy for the development of the highly effective low-toxicity ADAM17 inhibitors. In addition, as iRhom2 is a specific binding protein of ADAM17, targeting iRhom2 to inhibit ADAM17 activity is also a trend in the development of ADAM17 inhibitors.

## Discussion

Metalloproteinase ADAM17 holds a vital role in post-translational protein modification, gene transcription and post-transcriptional regulation, and is closely associated with tumors and inflammation. ADAM17 regulates cell membrane protein shedding and subsequent signal transduction. It can also be impacted by the interacting proteins and thus participate in the regulation of its downstream signaling pathways. ADAM17 has been implicated in immune regulation of tumor development. However, its immunomodulatory functions and mechanisms in cancer diseases are not well studied, and therefore more studies are needed to further determine the role of ADAM17 in tumor development. In this article, we summarized the structure and multiple regulatory roles of ADAM17, the latest immune regulation of ADAM17 in tumor formation and development, as well as the progress in the development of ADAM17 inhibitors. On the one hand, although the regulatory effect of ADAM17 on macrophages, NK cells, and endothelial cells has been confirmed in tumor, more key proteins or genes related to ADAM17 need to be identified, and the immune response involved in TME needs to be further explored. In addition, the role of ADAM17 in post-translational modifications, such as proteolysis, phosphorylation, glycosylation, and post-transcriptional regulation in cancer progression remains unclear. On the other hand, due to the structural homology of ADAM17’s catalytic domain with other metalloproteinases, more three-dimensional crystal structures associated with ADAM17 need to be uncovered to make them more conducive to highly selective and toxic drug design for ADAM17 inhibitors. How to reduce or avoid the toxic side effects of ADAM17 is also a potential research direction. Therefore, studying the key role and immunomodulatory mechanisms of ADAM17 in tumor development will provide new strategies for the prevention, diagnosis and treatment of cancer diseases.

## Author contributions

KW and HW conceived and designed this work. KW drafted the manuscript. KW and ZX edited and revised the manuscript. CY, XL, and MZ provided constructive suggestions for the manuscript and literature. All authors contributed to the article and approved the submitted version.

## Funding

This work was financially supported by the Research Funding of Luzhou Science and Technology Bureau (2021-JXJ-55; 41/00140011) (KW) and Support Funding of Southwest Medical University (41/00190026) (KW), Start-Up Research Funding of Southwest Medical University (41/00040179) (KW), and Research Funding of Southwest Medical University (41/00160216; 2021ZKQN108) (KW).

## Acknowledgments

We thank all the participants for their careful revision and intense discussion of the manuscript, and especially thank Prof. Asaduzzaman Khan and Prof. Yongxue Liu for their critical review for the manuscript.

## Conflict of interest

The authors declare that the research was conducted in the absence of any commercial or financial relationships that could be construed as a potential conflict of interest.

## Publisher’s note

All claims expressed in this article are solely those of the authors and do not necessarily represent those of their affiliated organizations, or those of the publisher, the editors and the reviewers. Any product that may be evaluated in this article, or claim that may be made by its manufacturer, is not guaranteed or endorsed by the publisher.
